# Assessment of Genetic Diversity, Runs of Homozygosity, and Signatures of Selection in Tropical Milking Criollo Cattle Using Pedigree and Genomic Data

**DOI:** 10.3390/genes13101896

**Published:** 2022-10-19

**Authors:** Ricardo Martínez-Rocha, Jorge Hidalgo, Alberto Cesarani, Rodolfo Ramírez-Valverde, Rafael Núñez-Domínguez, José Guadalupe García-Muñiz, Joel Domínguez-Viveros

**Affiliations:** 1 Facultad de Estudios Superiores Cuautitlán, Universidad Nacional Autónoma de México, Mexico 54740, MX, Mexico; 2 Department of Animal and Dairy Science, University of Georgia, Athens, GA 30602, USA; 3 Dipartimento di Agraria, University of Sassari, 07100 Sassari, Italy; 4 Departamento de Zootecnia, Posgrado en Producción Animal, Universidad Autónoma Chapingo, Chapingo 56230, MX, Mexico; 5 Facultad de Zootecnia y Ecología, Universidad Autónoma de Chihuahua, Chihuahua, Mexico 31453, CH, Mexico

**Keywords:** creole cattle, inbreeding, probability of gene origin

## Abstract

The objective of this study was to evaluate the genetic diversity of the Tropical Milking Criollo cattle (TMC) breed in Mexico through parameters derived from pedigree and genomic information assessment. The pedigree file consisted of 3780 animals. Seventy-nine bovines were genotyped with the medium-density single nucleotide polymorphism chip and considered a reference population for pedigree analysis. The effective population size and the probability of gene origin used to assess the evolution of genetic diversity were calculated with pedigree information. Inbreeding coefficients were evaluated based on pedigree ($F_{Ped}$), the genomic relationship matrix ($F_{GRM}$), and runs of homozygosity ($F_{ROH}$) of different length classes. The average inbreeding was 2.82 ± 2.66%, −0.7 ± 3.8%, and 10.9 ± 3.0% for $F_{PED}$, $F_{GRM}$, and $F_{ROH}$, respectively. Correlation between $F_{PED}$ and $F_{ROH}$ was significant only for runs of homozygosity > 4 Mb, indicating the $F_{PED}$ of a population with an average equivalent complete generation of five only recovers the most recent inbreeding. The parameters of the probability of gene origin indicated the existence of genetic bottlenecks and the loss of genetic diversity in the history of the TMC cattle population; however, pedigree and genomic information revealed the existence of current sufficient genetic diversity to design a sustainable breeding program.

## 1. Introduction

Genetic diversity refers to the heritable variation observed between and within populations, and it can indicate the degree of differentiation between or within any species, breed, or livestock population. Whether natural or artificial, population evolution relies primarily on the existing genetic diversity. A long history of evolutionary processes such as migration, mutation, selection, genetic drift, and adaptation, together with domestication, has created an enormous variety of breeds [1]. Locally adapted breeds have passed through these changes in harsh environments and probably developed specific adaptive features.

Cattle were introduced to America five centuries ago; these original introductions provided the genetic background of the American Criollo cattle, with influences from Spanish, Portuguese, and African breeds [2]. Tropical Milking Criollo (TMC) is a tropically adapted breed developed due to the geographic isolation of the Criollo cattle and farmers’ selection of milk production traits [3]. In Mexico, a nucleus herd of TMC cattle was formed in the mid-20th century. The base of this nucleus herd were cows imported from Nicaragua, creole cows from Mexico, and some bulls from the *Centro Agronómico Tropical de Investigación y Enseñanza* (CATIE), Turrialba, Costa Rica [4].

The knowledge of an animal population’s genetic diversity can help decide the breeding management for genetic improvement or conservation programs. Furthermore, the maintenance of genetic diversity in small populations is vital because heterozygosity and allelic diversity can be lost at an accelerated rate when these populations are closed and under artificial selection [5]. Additionally, the assessment of genetic diversity is a crucial step in understanding the evolutionary history of a breed since it provides essential information for the conservation and management of its biodiversity [6].

Population structure and genetic diversity can be assessed using pedigree information. Pedigree analysis is an important tool to describe genetic variability and its evolution across generations; however, its results heavily depend on the integrity of the pedigree [7]. SNP genotyping is one of the best ways to evaluate genetic diversity because of its availability, accuracy, and cost-effectiveness in livestock species [8].

The genetic variability of TMC cattle in Mexico has only been assessed by pedigree analysis [9]. Therefore, the objective of this study was to evaluate the genetic diversity of the TMC cattle breed population in Mexico through parameters derived from pedigree analysis and the assessment of the genome-wide single nucleotide polymorphism (SNP) genotyping array.

## 2. Materials and Methods

Genealogical and genotyping data were provided by the *Asociación Mexicana de Criadores de Ganado Romosinuano y Lechero Tropical* (AMCROLET). The pedigree file consisted of 3780 records (546 males and 3234 females) of animals born between 1956 and 2021. Seventy-nine bovines (5 males and 74 females) born between 2003 and 2018 were genotyped with the medium-density Affymetrix chip (63K SNP markers). These animals were selected for this study because they belong to a nucleus herd of the breed in Mexico. Quality control included a call rate ≥ 0.90 for SNP and animals. SNP and animals that did not satisfy this quality criterion were excluded, as well as those mapped on sexual chromosomes or not mapped on the Bos Taurus Autosome (BTA) 3.1.1 release. SNP were not filtered out for low minor allele frequency since this is a single-breed study, and we wanted to detect homozygosity correctly [10].

### 2.1. Pedigree Analyses

The pedigree analyses were performed using the ENDOG 4.8 program [11]. All analyses were performed for the total individuals in the pedigree and reference population, which consisted of the genotyped animals.

The inbreeding coefficient for each animal was estimated using the algorithm of Meuwissen and Luo [12]. The inbreeding coefficient determines the probability that two alleles at any locus are identical by descent. The average inbreeding coefficient among all animals ($F_{PED}$), percentage of inbred animals, and average inbreeding coefficient for inbred animals were calculated. The average relatedness coefficient (AR) was also estimated. This coefficient is computed as the probability that an allele randomly selected from the entire population (included in the pedigree) belonged to a particular animal [11].

The average equivalent complete generations, average maximum generations, and pedigree completeness index (PCI) by generation were used as indicators of the pedigree’s integrity. The equivalent complete generations of an individual were calculated as follows [13]:

$$EqG_{i}=\overset{k}{\underset{j=1}{\sum }}{\left(\frac{1}{2}\right)}^{n_{j}}$$

where n is the number of generations separating the individual i from his known ancestor j. The PCI is the mean proportion of ancestors known in each ancestral generation, and it was computed as the proportion of known ancestors in each ascending generation.

The interval generations (parent’s average age when their progeny, kept for future reproduction, were born) were calculated for the four following genetic pathways: sire of sire ($L_{SS}$), sire of dam ($L_{SD}$), dam of sire ($L_{DS}$), and dam of dam ($L_{DD}$). The average generation interval was computed as follows:

$$GI=\frac{L_{SS}+L_{SD}+L_{DS}+L_{DD}}{4}$$

The evolution of the genetic diversity in the population was assessed based on the probability of gene origin. The effective numbers of founders and ancestors were computed. The effective number of founders is defined as the number of founders contributing equally to produce the same genetic diversity in the evaluated population, and it was calculated according to Lacy [14]:

$$Fe=\frac{1}{\sum_{i=1}^{n}q_{i}^{2}}$$

where $q_{i}$ is the expected proportional genetic contribution of the founder i computed as the average relationship of that founder to each animal in the population; and n is the number of founders. The effective number of ancestors is the minimum number of ancestors (founders or not) necessary to explain the genetic diversity of the population, and it was calculated according to Boichard et al. [15]:

$$Fa=\frac{1}{\sum_{j=1}^{m}p_{i}^{2}}$$

where $p_{i}$ is the marginal contribution of the ancestor j computed as the genetic contribution not explained by other ancestors, and m is the number of ancestors.

The effective population size was estimated based on the individual increase of the inbreeding coefficient ($\Delta F_{i}$), calculated according to Gutierrez et al. [16]:

$$\Delta F_{i}=1-\sqrt[{Geq_{i}-1}]{1-F_{i}}$$

where Geq is the number of equivalent generations and F is the inbreeding coefficient for the individual i. The effective population size was estimated as:

$$N_{e}=\frac{1}{2\bar{\Delta F}}$$

### 2.2. Genomic Analyses

Individual inbreeding coefficients were calculated from the genomic relationship matrix ($F_{GRM}$) and runs of homozygosity ($F_{ROH}$). The $F_{GRM}$ were obtained by subtracting one from the diagonal elements of the genomic relationship matrix (G), built according to Yang et al. [17] using CTA v1.93.2 (Genome-wide Complex Trait Analysis) software. Runs of homozygosity (ROH) are long stretches of homozygous genotypes in an individual’s genome [18]. ROH were calculated separately for every animal by applying a sliding window of 20 SNP computed using the R package “DetectRuns” [19]. The minimum density considered and the maximum gap between consecutive SNP were 1 SNP every 1000 kb and 1 Mb, respectively. The $F_{ROH}$ was computed following the method proposed by McQuillan et al. [20]:

$$F_{ROH}=\frac{L_{ROH}}{L_{AUT}}$$

where $L_{ROH}$ is the total length of all the ROH detected in the animal’s autosomes, $L_{AUT}$ is the entire length of the autosomal genome. The $F_{ROH}$ by chromosome ($F_{ROH_{CHR}}$) also were calculated as the total length of all the ROH of the chromosome divided by the total length of the chromosome. Additionally, the ROH in the population were categorized into five length classes (1–2 Mb, 2–4 Mb, 4–8 Mb, 8–16 Mb, and >16 Mb) to analyze the distribution of the $F_{ROH}$ across these.

Selection signatures studies aim to identify genomic regions with a systematically reduced variation compared to the average across the genome. This idea has been implemented with ROH, searching for continuous parts of the genome without heterozygosity in the diploid state, and used on a genome-wide scale to detect signals of past selection [21]. The percentage of SNP existing within an ROH was estimated by counting the number of times each SNP appeared in an ROH and dividing that number by the number of animals [22]. This percentage had to be higher than 50% to signify a potential hotspot of ROH in the genome [23]. Genomic regions detected were interrogated for genes annotated to the Bos taurus genome assembly ASR-UCD1.2 using Genome Data Viewer (NCBI; https://www.ncbi.nlm.nih.gov/assembly/GCF_002263795.1/ accessed on 17 October 2022).

## 3. Results

### 3.1. Pedigree Analyses

The average *F_PED_* was 1.73% in the total population and 2.82% in the reference population (i.e., genotyped animals only). Inbred animals were 37.3% of the total population and 86.84% of the reference population. The average *F_PED_* for the inbred animals was 4.62% in the total population and 3.25% in the reference population. The *F_PED_* estimations in the total and reference populations suggest a low inbreeding level in the TMC cattle population. The mean AR estimated in the total and reference population were 2.75% and 4.67%, respectively.

**Figure 1:**
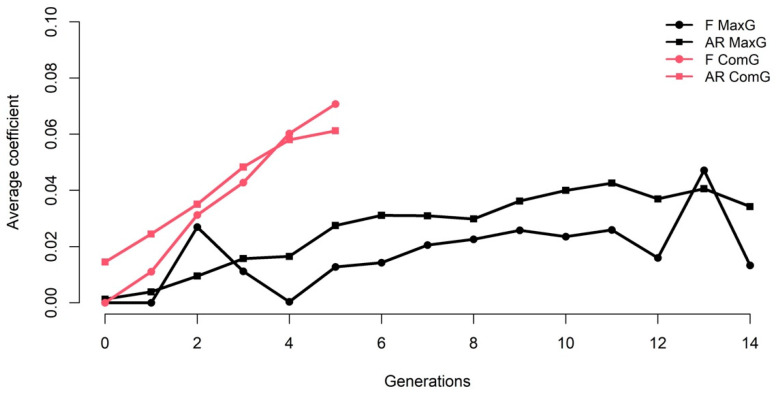
Evolution of the average pedigree-based inbreeding (F) and relatedness (AR) coefficients through complete (ComG) and maximum (MaxG) generations traced.

The realized effective population size based on the individual increase of *F_PED_* was 94.24, and the individual increase of *F_PED_* was 0.53%. The evolution of F and AR across complete generations traced and by the maximum number of generations traced are illustrated in Figure 1.

The average equivalent complete generations were 3.0 in the total population and 5.01 in the reference population. The average maximum generations were 7.3 in the total population and 10.4 in the reference population. The PCI one generation ago was 74.1% and 96.7% in the total and reference populations, respectively.

The PCI is an essential indicator of the *F_PED_* quality because it represents the harmonic mean of the parental genetic contributions. It is zero if one of the parents is unknown regardless of how deep and complete the pedigree of the other parent is [7].

**Figure 2:**
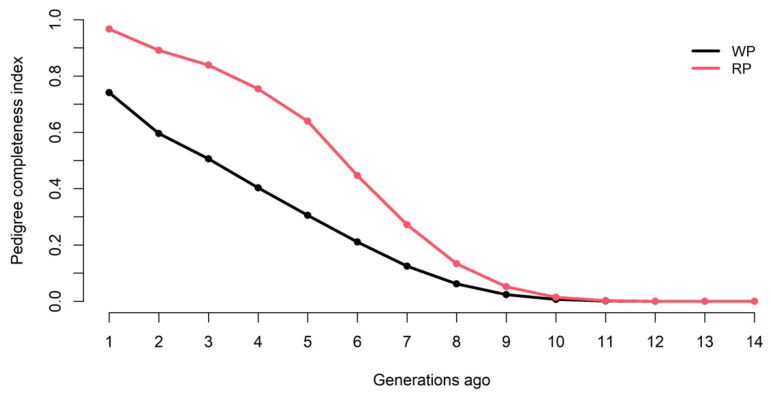
Pedigree completeness index in the whole (WP) and reference (RF) populations.

Five generations back, the PCI declined to 30.5% in the total population and 54.3% in the reference population (Figure 2).

The average generation interval for the total population was shorter than seven years (Table 1). The largest generation interval was for the path sire–sire, which means breeders take longer to select sires than dams.

The *Fe* was 78 and 45, whereas the *Fa* was 48 and 24 in the total and reference populations, respectively. A total of 19 and 9 ancestors explained 50% of the genetic diversity in the total and reference populations, respectively. The *Fa*/*Fe* ratio was 0.61 in the total population, while it was 0.53 in the reference population, indicating the existence of genetic bottlenecks and loss of genetic diversity in the TMC cattle population.

### 3.2. Genomic Analyses

The average *F_GRM_* was −0.68%, and the average *F_ROH_* (*L_ROH_* > 1 Mb) was 10.96% (Table 2).

*F_ROH_* represents a direct and correct estimate of the levels of homozygosity. ROH mainly reflects regions that are identical by descent on the genome, considering that all individuals are, to some extent, related if their ancestry is traced back far enough. Summary statistics of ROH identified across different length classes are reported in Table 3. A total of 9512 ROH > 1 Mb were detected, of which 76% had a length < 2 Mb.

**Figure 3:**
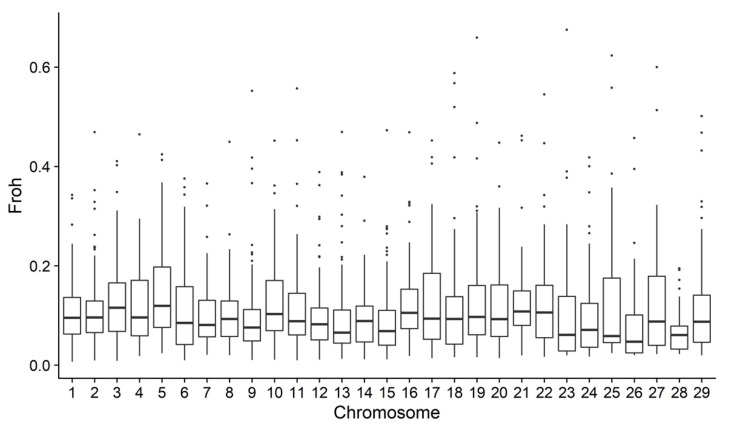
Runs of homozygosity (ROH) based inbreeding in each considered autosome in Tropical Milking Criollo cattle.

The *F_ROH_* varied across the autosomes (Figure 3). The mean of $F_{ROH\mathrm{Chr}}$ was 11.26%, the lowest average $F_{ROH\mathrm{Chr}}$ was found in BTA28, and the greatest average $F_{ROH\mathrm{Chr}}$ was estimated for BTA 5.

The correlations and their significance between all inbreeding coefficients estimated in this research are presented in Table 4. All the correlations involving *F_GRM_* were not significant, as well as the ones between $F_{PED}$ and $F_{GRM}$, $F_{ROH}$, $F_{ROH>1\;\mathrm{Mb}},$ and $F_{ROH>2\;\mathrm{Mb}}$. $F_{PED}$ and $F_{ROH>4\;\mathrm{Mb}}$ had a significant positive correlation (0.26–0.33).

**Figure 4:**
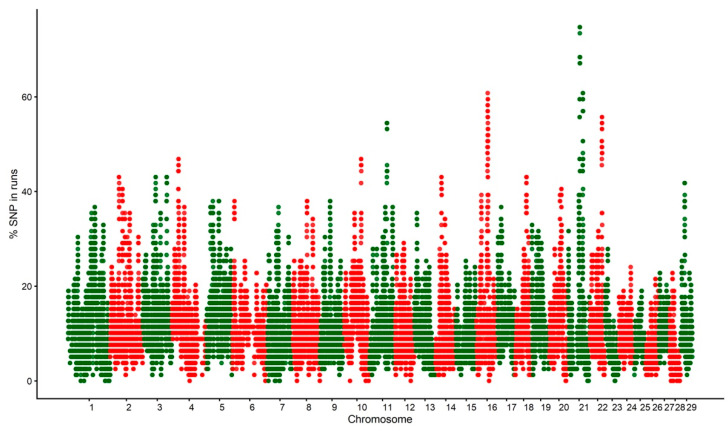
Manhattan plot of the SNP frequency within runs of homozygosity by autosome in the Tropical Milking Criollo cattle population.

Figure 4 shows the distribution of the percentage of SNP in ROH. Four ROH islands were found on chromosomes 11, 16, 21, and 22, using the 50% threshold (Table 5). Within the ROH islands found in this study, 126 genes were mapped.

## 4. Discussion

The differences between $F_{ROH}$ and $F_{PED}$ estimates suggest underestimation of $F_{PED}$ due to the shallow and missing pedigrees. The reference population, a sample of the actual TMC herds in Mexico, had a greater $F_{PED}$ and AR than the total population, meaning a loss of genetic diversity. When the AR is greater than $F_{PED}$, the frequency of mating between related animals is greater than between unrelated individuals [7]. In the last generation, considering the whole population, $F_{PED}$ was greater than AR, suggesting the frequency of mating between unrelated individuals was greater than the frequency of mating between related animals, as an effort to minimize the increase of the inbreeding level in this population.

Franklin [24] proposed the classic 50/500 recommendation for the minimum effective population size required to preserve adequate levels of genetic diversity in a population in the short/long term. The value Ne = 50 was derived for animal breeding programs, suggesting a maximum tolerable increase of inbreeding of 1% per generation to maintain genetic diversity at an acceptable level [25]. In our study, the estimated Ne (94.24) based on $F_{PED}$ is above the minimum tolerable in the short term; however, it is lower than the minimum Ne suggested for survival in the long term; thus, strategies to increase genetic diversity in the TMC cattle population are pertinent.

Hidalgo et al. [7] investigated the genetic diversity using pedigree and genomic analysis in Mexican Romosinuano cattle. They reported similar parameters of the probability of gene origin (*Fe* = 71; *Fa* = 31; *Fa*/*Fe* = 0.44) with a pedigree file of 4875 animals. Larger estimates related with the probability of gene origin (*Fe* = 113–541; *Fa* = 33–254; *Fa*/*Fe* = 29.2–61.5) were reported for six cosmopolitan beef cattle raised in Mexico [26].

The decrease in the effective population size and the loss in genetic diversity within a breed are usually due to increased mating between relatives because of a limited number of breeding animals. This is generally accompanied by an increase in the average inbreeding level of offspring and, the whole population. This scenario is common in populations under strong artificial selection, where the number of breeding animals is limited by human choices, or in small close populations, where natural constraints limit the number of breeding animals.

$F_{GRM}$ is based on the variance of additive genetic values and provides a measure relative to frequencies of the reference alleles in the base population. The $F_{GRM}$ estimated is sensitive to allele frequencies. In fact, $F_{GRM}$ by Yang’s method ranges from −1 to ∞ [27]. This means that $F_{GRM}$ can indicate some variability has been gained, but this gain can never be greater than 100% of the initial variability. It also means that $F_{GRM}$ can indicate a loss of variability higher than 100%. Zhang et al. [28] reported a mean $F_{GRM}$ of three dairy cattle breeds: in that study, Jersey cattle also had a negative value of $F_{GRM}$.

On the contrary, the $F_{ROH}$ does not depend on the allele frequencies. Therefore, estimators based on ROH from genotypes reflect homozygosity at the genome level and have the advantage of not being affected by allele frequency estimates or the pedigree’s incompleteness [28]. The length of an ROH can also be a valuable indicator of the time of the inbreeding event with which it is associated. Long ROH are related to recent events of inbreeding in the history of a breed or of a single individual, whereas short ROH indicate a more ancient event [29]. Most of the homozygosity in the TMC cattle population was found in ROH < 2 Mb length. This indicates that most of the inbreeding found in the TCM cattle population is old and might not have significant adverse effects (inbreeding depression) as deleterious alleles could be purged. However, some of the short-length ROH are likely to be false positives when detected using a medium-density SNP BeadChip, which leads to an overestimation of short-length ROH (<5 Mb) because of the high distance between adjacent SNP [30,31].

The longer ROH segments are, the more likely that recent inbreeding occurred within a population [29]. A pedigree with an average equivalent complete generation of five in the genotyped animals could be a limitation for detecting the presence of more ancient relatedness. The genes found within the ROH islands involve many molecular functions, biological processes, and cellular components. The ROH island in BTA22 was reported to be associated with age at first calving [32]. The *DCAF1* gene has been related to the hydroxymethylation of genomic DNA to maintain oocyte survival. The *HEMK1* plays a role in the development of the immune system, depending on environmental stressors [33]. Genes in the ROH island on BTA21 (*SNUPN*, *PTPN9,* and *CSPG4*) have been associated with the marbling score in Korean cattle [34]. The *PIK3CD* and *SPSB1* genes (both mapped on BTA16) have been related to immune response and regulation, respectively [35]. The *PEX14* gene on BTA16 is a candidate gene related to dairy production, which is under selection in dairy Holstein cattle [36]. The ROH island on BTA11 harbors important genes previously reported to be involved in reproductive traits, such as the *RBKS* gene associated with oocyte maturation [37], *SLC30A3* [38], and *CAD* [39] described as estrogen receptors, and *EMILIN1* associated with placentation and trophoblast invasion in the uterine wall [40].

## 5. Conclusions

The inbreeding coefficient estimates based on homozygosity runs in the Mexican Tropical Milking Criollo cattle population were larger than the estimates based on the pedigree and genomic relationship matrix. Most ROH had a length of less than 4 Mb, indicating ancient inbreeding. The correlation between $F_{PED}$ and $F_{ROH}$ was significant when the ROH were greater than 4 Mb, meaning that only long ROH, associated with recent inbreeding events in the breed’s history, are captured by the available pedigree records. The completeness of the pedigree was not sufficient to show more ancient inbreeding. Selection signatures were detected through highly homozygous regions related to reproductive and dairy production traits. Although there is evidence of past genetic bottlenecks and loss of genetic diversity, the Tropical Milking Criollo cattle population exhibits a reasonable amount of genetic diversity to design a sustainable breeding program.

## Figures and Tables

**Table 1:** Average generation intervals (±SE) computed for the four selection paths in Tropical Milking Criollo cattle.

Path of Selection	N	Average Generation Interval ± SE (y)
Sire–Sire	187	7.45 ± 0.42
Sire–Dam	957	7.31 ± 0.16
Dam–Sire	183	6.94 ± 0.26
Dam–Dam	827	6.46 ± 0.12
Total	2154	6.97 ± 0.09

**Table 2:** Different inbreeding coefficients based on genomic information ($F_{GRM}$ and $F_{ROH}$) ^1^ in the TMC cattle population.

	*F_GRM_*	$F_{ROH}$	$F_{ROH>1\;Mb}$	$F_{ROH>2\;Mb}$	$F_{ROH>4\;Mb}$	$F_{ROH>8\;Mb}$	$F_{ROH>16\;Mb}$
Mean ± SD (%)	−0.7 ± 3.8	10.9 ± 3.0	10.9 ± 3.0	6.0 ± 2.9	4.2 ± 2.6	3.1 ± 2.1	2.1 ± 1.1
Min (%)	−14.9	5.3	5.7	0.8	0.16	0.3	0.6
Max (%)	25.9	18.0	18.7	13.1	11.	8.4	5.2

^1^$F_{GRM}$ = inbreeding based on the genomic relationship matrix; $F_{ROH}$ = inbreeding based on runs of homozygosity.

**Table 3:** Number and mean length [in Mb and number of single-nucleotide polymorphisms (SNP)] of runs of homozygosity (ROH) in different length classes.

		Mean Length ± SD ^1^	
ROH Length Class	Number of ROH	Mb	SNP
1–2 Mb	7236	1.35 ± 0.26	28 ± 17
2–4 Mb	1444	2.66 ± 0.52	51 ± 28
4–8 Mb	453	5.55 ± 1.16	123 ± 56
8–16 Mb	268	11.28 ± 2.24	243 ± 70
>16 Mb	111	23.71 ± 7.21	465 ± 157
Total	9512	2.28 ± 3.06	48 ± 69

^1^ Total values were obtained as the average length of the total ROH (considering length > 1 Mb) and the average number of SNP in the ROH.

**Table 4:** Pearson’s correlations (above diagonal) among genomic- ($F_{GRM}$), pedigree- ($F_{PED}$), and Runs of Homozygosity- ($F_{ROH}$) based inbreeding coefficients; significance values ^1^ are reported below the diagonal.

	$F_{PED}$	$F_{GRM}$	$F_{ROH}$	$F_{ROH>1\;Mb}$	$F_{ROH>2\;Mb}$	$F_{ROH>4\;Mb}$	$F_{ROH>8\;Mb}$	$F_{ROH>16\;Mb}$
$F_{PED}$		0.11	0.17	0.17	0.19	0.26	0.29	0.33
$F_{GRM}$	ns		0.10	0.10	0.15	0.23	0.24	−0.02
$F_{ROH}$	ns	ns		1.00	0.96	0.91	0.85	0.62
$F_{ROH>1\;Mb}$	ns	ns	***		0.96	0.91	0.85	0.62
$F_{ROH>2\;Mb}$	ns	ns	***	***		0.97	0.91	0.63
$F_{ROH>4\;Mb}$	*	ns	***	***	***		0.95	0.65
$F_{ROH>8\;Mb}$	**	ns	***	***	***	***		0.70
$F_{ROH>16\;Mb}$	**	ns	***	***	***	***	***	

^1^ ns = non-significant; * = *p* < 0.05; ** = *p* < 0.01; *** = *p* < 0.001.

**Table 5:** Description of the runs of homozygosity (ROH islands) found in the autosomal genome of the Tropical Milking Criollo cattle population.

BTA	Start (bp)	End (bp)	n SNP	Genes
11	71,623,956	72,732,727	17	*BABAM2, RBKS, MRPL33, SLC4A1AP, SUPT7L, GPN1, CCDC121, ZNF512, C11H2orf16, GCKR, FNDC4, IFT172, KRTCAP3, NRBP1, PPM1G, ZNF513, SNX17, EIF2B4, GTF3C2, MPV17, UCN, TRIM54, DNAJC5G, SLC30A3, CAD, ATRAID, SLC5A6, TCF23, PREB, ABHD1, CGREF1, KHK, EMILIN1, OST4, AGBL5, TRNAA-AGC, TRNAY-GUA, TMEM214, MAPRE3, DPYSL5*
16	42,918,309	45,953,414	35	*PEX14, DFFA, CORT, CENPS, PGD, UBE4B, NMNAT1, CTNNBIP1, PIK3CD, TMEM201, SPSB1, MIR34A, CA6, ENO1, SLC45A1, LOC786597, ERRFI1, PARK7, TNFRSF9, VAMP3*
21	31,679,297	46,217,299	41	*RCN2, PSTPIP1, TSPAN3, HMG20A, ODF3L1, CSPG4, SNX33, IMP3, SNUPN, PTPN9, MAN2C1, NEIL1, MIR631, COMMD4, PPCDC, SCAMP5, COX5A, FAM219B, MPI, SCAMP2, ULK3, CPLX3, CSK, EDC3, CLK3, ARID3B, UBL7, SEMA7A, CYP11A1, STRA6, ISLR, PML, STOML1, LOXL1, GZMB, STXBP6, MIR2888-1, NOVA1, G2E3, SCFD1, COCH, STRN3, AP4S1, NUBPL, AKAP6, MIR6522, EGLN3, SPTSSA, EAPP, SNX6, CFL2, BAZ1A, LOC613444, SRP54, FAM177A1, PPP2R3C, KIAA0391, PSMA6, NFKBIA, INSM2, BRMS1L*
22	49,273,889	49,841,675	12	*RBM15B, MANF, MAPKAPK3, CISH, HEMK1, C22H3orf18*

## Data Availability

The dataset is not publicly available since it belongs to AMCROLET and is treated as confidential information.
